# Anesthetic Management of a 33-Year-Old Female With Limb-Girdle Muscular Dystrophy Undergoing Laparoscopic Cholecystectomy: A Case Report

**DOI:** 10.7759/cureus.111852

**Published:** 2026-06-30

**Authors:** Nezar Gargori, Atheer A Alboloshi, Abrar Nawawi, Sara Farsi

**Affiliations:** 1 Anesthesiology, King Abdulaziz University Hospital, Jeddah, SAU; 2 Medicine and Surgery, Faculty of Medicine, King Abdulaziz University, Jeddah, SAU; 3 Surgery, King Abdulaziz University Hospital, Jeddah, SAU; 4 Anesthesia, King Abdulaziz University Hospital, Jeddah, SAU

**Keywords:** anesthesia, case report, laparoscopic cholecystectomy, limb-girdle muscular dystrophy, total intravenous anesthesia

## Abstract

Limb-girdle muscular dystrophy (LGMD) is a heterogeneous group of rare neuromuscular disorders characterized by progressive muscle weakness in the hip and shoulder muscles. Like many other muscular dystrophies, LGMD patients are at increased risk of rhabdomyolysis, malignant hyperthermia (MH), and cardiopulmonary compromise, posing significant anesthetic challenges for these patients. Despite this, there are few reports on anesthesia management in patients with LGMD. We present the anesthetic management of a 33-year-old single female with LGMD undergoing laparoscopic cholecystectomy.

A 33-year-old Middle Eastern, wheelchair-dependent female with LGMD (subtype 2A) presented for elective laparoscopic cholecystectomy following recurrent biliary colic. Preoperative evaluation revealed normal cardiac function and minimal pulmonary involvement. Total intravenous anesthesia (TIVA) with propofol, fentanyl, rocuronium, and morphine was administered without volatile anesthetics. Intraoperative monitoring followed the American Society of Anesthesiologists standards. The surgery was uneventful, and the patient was extubated successfully without ICU admission. General anesthesia with TIVA is considered a safe approach for LGMD patients undergoing laparoscopic surgery. Avoidance of triggering agents and meticulous perioperative planning are essential to minimize complications.

## Introduction

Limb-girdle muscular dystrophy (LGMD) encompasses a heterogeneous group of inherited myopathies, with type 2A being the most common. It is characterized by progressive weakness of the pelvic and shoulder girdle muscles [1,2]. Clinically, LGMD typically presents in late childhood to early adulthood with insidious onset of proximal lower-extremity weakness, such as difficulty climbing stairs, rising from a squat, or frequent falls, followed by progressive involvement of the shoulder girdle, manifesting as trouble lifting objects or combing hair. Physical examination often reveals a waddling gait, scapular winging, and a positive Gowers' sign, while facial, bulbar, and distal muscles are characteristically spared, helping to distinguish LGMD from other muscular dystrophies [1,2]. Patients with LGMD face significant anesthetic challenges due to increased risks of respiratory insufficiency, cardiomyopathy, rhabdomyolysis, and potential susceptibility to malignant hyperthermia [3-5]. Furthermore, they may exhibit exaggerated and unpredictable responses to neuromuscular blocking agents [3,6,7]. While the general principles of anesthetic management for neuromuscular disorders are established, literature specifically guiding the care of LGMD patients remains limited, and no formal consensus guidelines exist. This report describes the successful perioperative management of a 33-year-old female with genetically confirmed LGMD undergoing elective laparoscopic cholecystectomy. We highlight the safe and effective use of a rocuronium-sugammadex neuromuscular blockade strategy within a trigger-free, total intravenous anesthetic (TIVA) technique, which involves intravenous administration of all anesthetic agents and avoids volatile inhalational anesthetics that could trigger malignant hyperthermia [1,3]. This case adds to the growing evidence and provides a potential management option for similar high-risk patients undergoing laparoscopic surgery, demonstrating that with meticulous multidisciplinary planning, these patients can undergo necessary surgeries without requiring intensive care admission.

## Case presentation

A 33-year-old Middle Eastern female, 58 kg, 1.58 m, with genetically confirmed LGMD with calpain 3 deficiency, LGMD-2A/R1, diagnosed at age 18 years following progressive proximal muscle weakness manifesting as difficulty climbing stairs. Her weakness progressed gradually over the span of seven years until she became wheelchair-dependent a few months before the surgery. Family history was notable for an affected paternal uncle, while her four sisters and two brothers remain asymptomatic. The patient also suffers from migraine and is a carrier for biotinidase deficiency. Furthermore, one year earlier, she had an episode of acute cholecystitis for which she was hospitalized and treated conservatively. At that time, surgical intervention was deferred due to concerns about potential anesthetic complications related to her underlying LGMD. Since then, she had remained symptom-free until one month before her recent admission for cholecystitis, when she presented with right upper quadrant pain. Initially, she was admitted to another hospital and was diagnosed with chronic cholecystitis after an ultrasound was performed. The patient was admitted there for two weeks and was managed conservatively on antibiotics, proton pump inhibitor (PPI), analgesia, and enoxaparin. The patient was transferred to a higher center for continuation of care, given the nature of her chronic illness and the high risk for perioperative anesthesia-related complications.

Preoperative evaluation

The patient underwent a standard preanesthetic assessment, in addition to comprehensive cardiac and pulmonary evaluations. Cardiac evaluation included ECG, transthoracic echocardiography, and laboratory investigations, including cardiac troponin-I and pro-B-type natriuretic peptide (pro-BNP). Transthoracic echocardiography revealed normal biventricular function (left ventricular ejection fraction {LVEF} 57%) with trace tricuspid regurgitation and no structural abnormalities. ECG was unremarkable with normal laboratory readings of cardiac troponin-I <0 and pro-BNP <35 pg/mL, excluding cardiac manifestations of LGMD such as dilated cardiomyopathy [8]. Pulmonary evaluation included a pulmonary function test, which showed forced vital capacity (FVC) of 2.56 L and a forced expiratory volume in 1 second (FEV1)/FVC of 0.96, indicating possible restrictive lung disease, most likely due to respiratory muscle weakness given the LGMD history.

Laboratory investigations were conducted to establish baseline values and detect potential complications, such as rhabdomyolysis and hyperkalemia. Baseline readings were 3.12 kU/L for creatine kinase, 3.8 mmol/L for potassium, 2.29 mmol/L for calcium, and 2.7 mmol/L for blood urea nitrogen (BUN). Standard clinical and paraclinical assessments were unremarkable, including normal complete blood count and bleeding profile, Mallampati score of 1, and no history of difficult intubation. Additional preoperative workup included Doppler ultrasound of the legs due to the heightened risk of deep vein thrombosis given the patient's wheelchair-bound status.

Anesthetic management

The anesthetic protocol was designed with consideration for the patient's neuromuscular condition. Malignant hyperthermia precautions were implemented, including avoidance of all triggering agents, such as volatile anesthetics and succinylcholine. Thus, induction was achieved using total intravenous anesthesia (TIVA) with propofol (150 mg), fentanyl (100 μg), and rocuronium (50 mg), which was reversed by sugammadex (200 mg) at the end. Endotracheal intubation was achieved by placing the patient in reverse Trendelenburg position. Maintenance consisted of propofol infusion (12 mg/kg/h, which was decreased to 4.8 mg/kg/h toward the end of surgery), fentanyl (1-5 μg/kg/h), and dexmedetomidine (0.2 μg/kg/h) supplemented with morphine (3 mg) for analgesia and ondansetron (4 mg) plus dexamethasone (8 mg) for postoperative nausea and vomiting prophylaxis. After induction, we performed a lateral quadratus lumborum block on the right side + left lateral transversus abdominis plane block using bupivacaine 0.25%, 20 mL for each block. Comprehensive monitoring included continuous temperature and hemodynamic measurements (with heart rate maintained at 55-70 bpm and blood pressure at 120-138/70-85 mmHg) (Table 1).

**Table 1:** Anesthetic drugs and doses used.

Drug	Dose	Phase
Propofol	150 mg (induction); 12 mg/kg/h, 4.8 mg/kg/h (maintenance)	Induction and maintenance
Fentanyl	100 μg (induction); 1-5 μg/kg/h (maintenance)	Induction and maintenance
Rocuronium	50 mg	Induction
Bupivacaine 0.25%	20 mL (right lateral quadratus lumborum block) + 20 mL (left lateral transversus abdominis plane block)	Regional block (postinduction)
Dexmedetomidine	0.2 μg/kg/h	Maintenance
Morphine	3 mg	Maintenance
Ondansetron	4 mg	Maintenance
Dexamethasone	8 mg	Maintenance
Sugammadex	200 mg	Reversal (end of procedure)

After reversing the rocuronium with sugammadex and upon weaning the anesthetics, the patient showed signs reflecting satisfactory respiratory muscle function, where her tidal volume was 700 mL with a regular respiratory rhythm that was reassuring to extubate her on the table and send her to the postanesthesia care unit (PACU) and avoid high dependency care unit admission.

Postoperative course

Upon arrival in the PACU, the patient's initial vital signs were a blood pressure of 170/110 mmHg, heart rate of 67 bpm, and oxygen saturation of 99% on room air. The elevated blood pressure was attributed to acute postoperative pain. Hence, intravenous meperidine (20 mg) was administered to control early pain in the PACU. Postoperative analgesia was managed with scheduled paracetamol (1 g QID) and ibuprofen (400 mg every 8 h). The patient maintained stable respiratory and hemodynamic parameters postoperatively and did not require admission to the intensive care unit. Moreover, throughout the perioperative period, there were no signs of malignant hyperthermia (e.g., hyperthermia, tachycardia, hypercapnia, or metabolic acidosis). However, her hospital stay was prolonged due to gastric fullness and early satiety, which raised suspicion of gastroparesis, a known gastrointestinal manifestation of muscular dystrophy that can be exacerbated by surgical stress. She was placed on a soft diet with close monitoring to mitigate aspiration risk, given her pre-existing respiratory muscle weakness. Prophylactic respiratory therapy was also initiated to support pulmonary function and prevent atelectasis. Her symptoms gradually improved, and she was discharged on postoperative day four following a favorable recovery.

## Discussion

The anesthetic management of patients with LGMD shares similarities with other neuromuscular disorders, yet complications may arise unpredictably, even in those with mild or stable disease. Our case highlights several critical considerations for LGMD patients undergoing surgery.

Key anesthetic challenges in LGMD

Malignant Hyperthermia Risk

While LGMD is not definitively linked to malignant hyperthermia (MH) susceptibility, the theoretical risk of rhabdomyolysis and hypermetabolic reactions warrants caution [1]. Our patient received strict MH precautions, including avoidance of volatile anesthetics and succinylcholine. Notably, her uneventful course supports the safety of TIVA in LGMD, a finding consistent with prior reports. Specifically, a 36-year-old woman with LGMD 2B undergoing arthroscopic knee surgery and a 61-year-old man with LGMD undergoing aortic aneurysm repair both received TIVA without complications [1,9,10].

Respiratory and Airway Management

LGMD patients are predisposed to aspiration due to potentially decreased laryngeal reflexes and delayed gastric emptying time. Therefore, a longer preoperative fasting period may be considered on an individualized basis. Moreover, LGMD patients are also at heightened risk for masseter spasm and difficult airway [3]. Thus, we prepared for a difficult airway despite our patient’s Mallampati score of 1 and lack of prior intubation difficulties.

Neuromuscular Blockade (NMB)

The administration of rocuronium, successfully reversed with sugammadex, allowed early extubation in our patient, supporting the potential safety of using rocuronium as needed in LGMD [3,6,7]. Similar outcomes were reported in a 57-year-old woman undergoing total thyroidectomy and a 71-year-old man with difficult airway and global hypoventilation undergoing partial nephrectomy, both of whom received rocuronium-sugammadex without adverse events [3].

Cardiovascular Stability

Although our patient’s cardiac evaluation tests were normal, LGMD subtypes (e.g., 2C-2F) may involve cardiomyopathy or arrhythmias [4,5]. Thus, comprehensive preoperative cardiac evaluation is crucial for guiding individualized anesthetic management and ensuring hemodynamic stability. In our case, the patient maintained stable cardiac function throughout the perioperative period without arrhythmias or decompensation.

Strengths

A principal strength of this case is the demonstration of a comprehensive, multimodal anesthetic strategy that proactively addressed the major risks in LGMD. This included the consistent application of a trigger-free TIVA technique for MH prophylaxis, use of regional analgesia (right lateral quadratus lumborum block and left TAP block) to minimize opioid-related respiratory depression, and meticulous preoperative cardiopulmonary evaluation. These interventions facilitated immediate extubation and avoided ICU admission, underscoring the value of tailored, multidisciplinary planning for high-risk patients.

Limitations

The successful outcome described in this case offers a valuable management framework; however, its broader application should be considered according to the patient's context. This report is a single case with a specific LGMD subtype (type 2A/calpain-3 deficiency), and findings may not be directly generalizable to other LGMD subtypes or to patients with more advanced cardiorespiratory involvement. For example, our patient's well-preserved cardiorespiratory status was a favorable factor when considering rocuronium and sugammadex, but outcomes may differ in individuals with more advanced disease. Furthermore, train‑of‑four (TOF) monitoring was not available at our institution; therefore, neuromuscular blockade reversal was guided solely by clinical signs. Finally, the optimal dosing and monitoring of neuromuscular blockers in LGMD remain areas for future research.

## Conclusions

This case demonstrates that laparoscopic cholecystectomy under TIVA is a viable option for patients with LGMD, provided MH precautions, meticulous preoperative evaluation, and vigilant intraoperative monitoring. LGMD remains a heterogeneous condition, and anesthetic strategies should be tailored to individual risk profiles. Further studies are needed to refine anesthetic protocols for LGMD. In order to ensure maximum safety for patients during the perioperative period, we emphasize the importance of a comprehensive multidisciplinary preoperative evaluation.
